# Molecular Dynamics Simulations on the Thermal Decomposition of Meta-Aramid Fibers

**DOI:** 10.3390/polym10070691

**Published:** 2018-06-21

**Authors:** Fei Yin, Chao Tang, Qian Wang, Xiong Liu, Yujing Tang

**Affiliations:** 1 College of Engineering and Technology, Southwest University, Chongqing 400715, China; yf1992@email.swu.edu.cn (F.Y.); 18382427662@139.com (Y.T.); 2 Electric Power Research Institute of State Grid Chongqing Electric Power Company, Chongqing 401123, China; qianwang@126.com (Q.W.); lingli198967@126.com (X.L.)

**Keywords:** meta-aramid, thermal decomposition, ReaxFF, reactive molecular dynamics

## Abstract

The thermal decomposition mechanism of a meta-aramid fiber was simulated at the atomic level using the ReaxFF reactive force field. The simulation results indicated that the main initial decomposition positions of the meta-aramid fiber elements were C_aromatic ring_–N and C=O, which could be used as targets for the modification of meta-aramid fibers. The meta-aramid fiber elements first decomposed into C6–C13 and then into smaller segments and micromolecular gases. The temperature was shown to be the key factor affecting the thermal decomposition of the meta-aramid fibers. More complex compositions and stable gases were produced at high temperatures than at lower temperatures. HCN was a decomposition product at high temperature, suggesting that its presence could be used for detecting thermal faults in meta-aramid fibers. Generation path tracing of the thermal decomposition products NH_3_ and H_2_O was also performed. NH_3_ was produced when the NH_2_ group captured an H atom adjacent to the system. H_2_O was formed after a carbonyl group captured an H atom, became a hydroxyl group, with subsequent intramolecular dehydration or intermolecular hydrogen abstraction.

## 1. Introduction

As a new material with high mechanical strength, high temperature resistance, and excellent electrical properties, meta-aramid fiber is an aromatic polyamide that has been widely applied in transformer coils, windings, motor windings, line and cable wires, and other insulation components. Their excellent mechanical and electrical properties has also led to application of meta-aramid fibers in oil–paper insulation systems, as the operation state of these systems in a large oil-immersed transformer directly affects the safety of power transmission [1].

Many studies suggest that thermal fatigue is the primary factor affecting insulation paper aging. Deterioration of the mechanical properties of insulation paper occurs with aging of the oil–paper insulation material. Although the aramid fiber exhibits excellent mechanical properties and thermal stability, in the complex internal environment of a transformer (especially an ultra-high voltage power transformer), the comprehensive stresses of the thermal field, electric field, and mechanical stress will accelerate the aging rate of oil-immersed aramid insulation papers [1]. Chemical decomposition of meta-aramid fibers can be divided into three processes: oxidation, hydrolysis, and thermal cracking. Thermal decomposition of aramid-based insulation papers causes molecular chain fractures, a reduced polymerization degree, and the production of large quantities of water and gaseous HCN, NH_3_, CH_4_, and CO_2_ [2,3], posing a threat to the safe operation of the transformer.

Molecular simulation tools, including quantum chemistry, molecular mechanics, and molecular dynamics simulations, have gradually emerged with the development of computer technology and theoretical chemistry. Compared with quantum chemistry and molecular mechanics, the advantage of molecular dynamics is that the dynamic behavior of matter can be studied as a function of time at the atomic and molecular level. To investigate the formation and breaking of chemical bonds, different simulation methods (advanced sampling/biased simulation) have been successively proposed [4,5,6,7,8,9,10,11,12], including ab-initio molecular dynamics, the empirical valence bond (EVB) method, multi-surface adiabatic reactive molecular dynamics (MS-ARMD), the Monte Carlo method, density functional theory (DFT), and ReaxFF molecular dynamics. Van Duin and Dasgupta proposed the ReaxFF force field simulation tool in 2001. The fracture and formation of bonds between atoms in the force field are defined by the bond angle and torsional force as a function of the bond order and the relationships between bond length, bond order, and bond energy [12]. The reactive force field ReaxFF can be used to evaluate the dynamic process of chemical reactions to simulate the fracture and formation of chemical bonds and to simulate complex chemical reactions of large systems at a high simulation speed. Reactive molecular dynamics (RMD) has been successfully applied to organic micromolecular systems, polymers, metal oxides, and other new materials [13,14,15,16,17,18,19,20].

Beste [21] employed ReaxFF force field to study the oxidation process of lignin, describing the production of carbon fibers. Liu et al. [22] conducted an in-depth study on the initial stage reaction mechanism of a new combustion material at the beginning of high-temperature oxidation with ReaxFF force field, obtaining the chemical reaction network of the four components in an alternative fuel system. The ReaxFF force field was used to simulate the combustion of hydrogen and reveal the combustion mechanism of hydrogen [23]. Previous studies have simulated aging and decomposition of different substances using RMD based on the principle that molecular collision is enhanced by increasing temperature [24,25,26,27,28,29]. Rom et al. [24] simulated the initial thermal decomposition of nitromethane within a 2500–4500 K temperature range using the ReaxFF force field. The oxidation process of methane was studied using heat to determine the mechanism of partial oxidation [25]. The decomposition mechanism of transformer oil was simulated at 2400 K, 2600 K, and 3000 K by accelerating a heating reaction [26]. Using RMD Yan et al. [27] studied the decomposition process of insulation paper at 1500 K, 1700 K, and 1900 K. Researchers studied the influencing mechanism of water on insulation paper pyrolysis at 1600 K, 1800 K, and 2000 K using a reactive force field [28]. At 2000 K, simulation of the pyrolysis of a coal molecular model [29] revealed the pyrolysis mechanism of the complex system of coking coal and lignite.

Most studies on meta-aramid fibers have relied on macroscopic experimental methods. However, these experimental methods cannot be used to explain the complex phenomena of microreactions and the microscopic mechanisms. As a scientific calculation method, the molecular dynamics method can be used to clarify these previously unexplainable mechanisms [30,31,32,33,34,35,36]. Further investigation of the degradation mechanism and thermal decomposition of meta-aramid fiber at the micro-level using molecular simulation is needed. In this study, Materials Studio (MS) software (Accelrys, San Diego, CA, USA) was used to characterize the thermal decomposition micromechanism of meta-aramid fiber using a ReaxFF force field, providing theoretical support for thermal fault diagnosis as well as data for future research on meta-aramid fiber modifications.

## 2. Modeling

**Figure 1:**
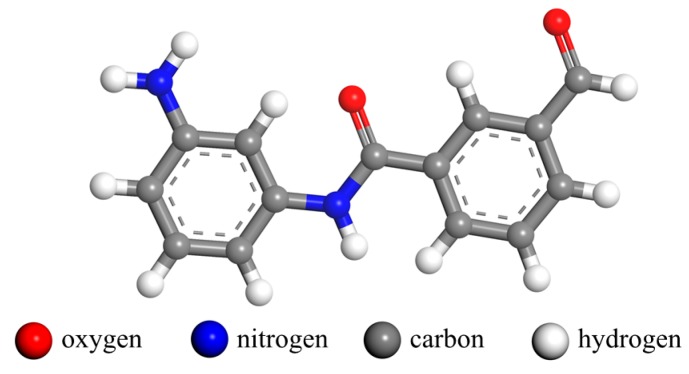
Molecular elemental structure of meta-aramid fiber.

First, the structural element molecule of the meta-aramid fiber was established using the MS software, and then a meta-aramid fiber amorphous model was constructed using the Amorphous Cell module. To study the case of initially broken bonds among meta-aramid fiber elements, a model including three molecules (3-PMIA) was established. To study and analyze the main products and generation paths of the meta-aramid fiber thermal decomposition process, a model including 30 molecules (30-PMIA) was also established. The simulations were repeated. When the model was constructed, the boundary condition was set as the periodic boundary condition. The initial density was set as 1.0 g/cm^3^. The cell size of the 3-PMIA model was 10.62 × 10.62 × 10.62 Å^3^ with 90 atoms in the model, and the cell size of the 30-PMIA model was 22.87 × 22.87 × 22.87 Å^3^ with 900 atoms in the model. The molecular elemental structure of the meta-aramid fiber is shown in Figure 1.

Previous studies have shown that the pyrolysis of meta-aramid fiber occurs at approximately 673 K [37,38]. The decomposition process of meta-aramid fiber simulated by molecular dynamics indicates that no pyrolysis will occur at a low temperature within a short period. Therefore, in this study, a previously studied and widely accepted method was applied, and the simulation temperature was increased to speed up the reaction rate to achieve an accelerated aging process [39,40]. The decomposition reaction of the meta-aramid fiber was slow at low temperature but fast at high temperature, which was unfavorable for observation of the reaction path. After repeated simulations, the temperature range of 2000 K–3000 K was considered suitable. To study the effect of temperature on the pyrolysis products, the simulation temperatures were set as 2000 K, 2300 K, 2500 K, 2700 K, and 3000 K.

**Figure 2:**
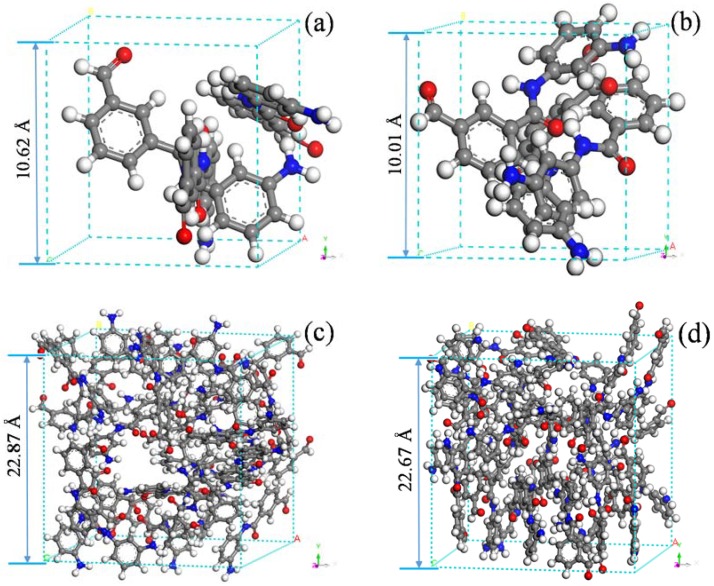
Models of meta-aramid fiber before and after optimization: (**a**) initial 3-PMIA model, (**b**) optimized 3-PMIA model, (**c**) initial 30-PMIA model, (**d**) optimized 30-PMIA model.

To ensure the balanced state of the constructed model and obtain a simulation system with a rational configuration, structural optimization of the meta-aramid fiber model was first performed using 5000 structural iterative steps. An annealing cycle treatment was then performed in the temperature range of 300 K–1000 K. A 500-ps relaxation process was then performed. Following these steps, a stable meta-aramid fiber structure was obtained. The Dreiding force field was adopted in the model construction and relaxation process [22,41]. Repeated simulation results revealed almost no difference in the densities of the models before and after optimization at approximately 1.35 g/cm^3^. The initial and optimized models are shown in Figure 2. The NVT ensemble (with a certain particle number *N*, volume *V*, and temperature *T*) was adopted in this paper, the ReaxFF force field was applied in the dynamic (RMD) simulation, the time step was set as 0.1 fs, the simulation track file was collected every 0.1 ps, and the simulation time was 100 ps. A Nosé temperature regulator was used for temperature control, and a Berendsen pressure regulator was used for pressure control [42,43].

## 3. Analysis of Simulation Results

### 3.1. Position of Initially Broken Bonds

In this paper, the statistical results at 2000 K are taken as an example to analyze the position of the initial break position, and the number of fractures for the initially broken bonds are listed in Table 1. The bond fractures were mainly of the N–H bond on the amide group and the detachment of the H atom (dehydrogenation reaction) on the benzene ring. The number of N–H bond fractures was much less than the number of detachments of the H atom on the benzene ring because of the formation of a large number of hydrogen bonds, which reduces the fractures of other chemical bonds. In the early decomposition stage of the meta-aramid fiber, the fracture of N–H bonds on the amide group and the detachment of the H atom on the benzene ring were only slightly affected by the mechanism of the meta-aramid fiber. Hence, no statistical analysis was performed.

The results in Table 1 indicate that the most common initially broken position on the main chain was at the C_aromatic ring_–N(C_ar_–N) bond, which accounted for 50% of the fractures. This result indicates that C_ar_–N bonds are a weak link in the meta-aramid fiber and play an important role in the initial decomposition. The fracture of the C_ar_–N bond will not only reduce the chemical stability of the meta-aramid fiber, but also the thermal stability. In addition, the C=O double bond broke many times on the main chain of the meta-aramid fiber. This position could be used as a specific target for material modification. The time tracing of chemical bond fracture during the initial reaction revealed that, at high temperature, pyrolysis by the detachment of benzene-ring hydrogen atoms (dehydrogenation reaction) occurs first, followed by fracture of the C_ar_–N bond. The fracture of C=O and other bonds occurred later. At the same time, the broken bond on the main chain of meta-aramid fibers also occurred in C_aromatic ring_–C_carbonyl_ and C_amide group_–N_amide group_.

**Figure 3:**
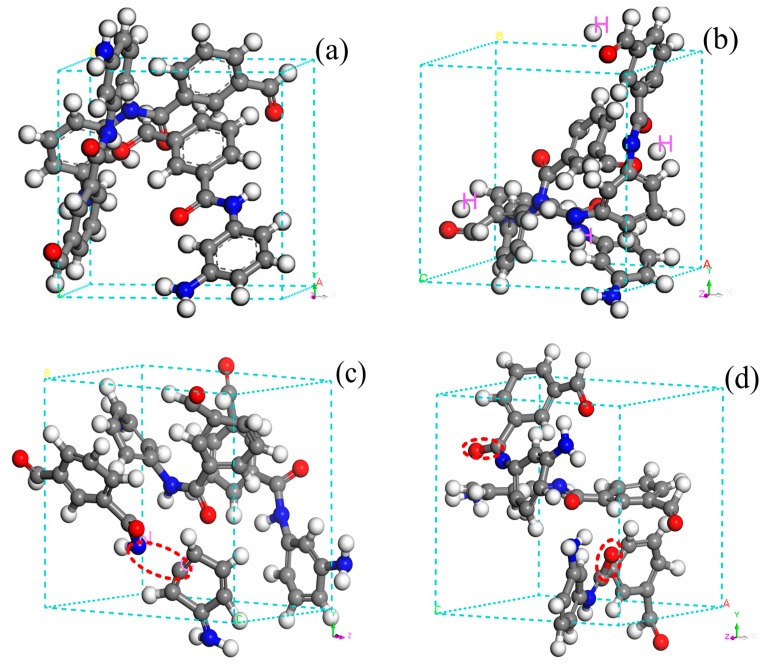
Chemical bond fracture behavior for the initial reaction: (**a**) initial model, (**b**) dehydrogenation reaction, (**c**) C_aromatic ring_–N bond fracture, and (**d**) C=O bond fracture.

Therefore, the fracture of the molecular chain C_ar_–N bond occurs during the early stage of the pyrolysis reaction, which generates macromolecule-sized chain segments. By continuing pyrolysis, the benzene ring on the main chain of the meta-aramid fiber will open, producing micromolecular fragments, gases, and free radicals. This result agrees well with that of a previous study [2]. The bond fracture behavior during the initial reaction is shown in Figure 3.

### 3.2. Kinetic Calculation of Meta-Aramid Fiber Pyrolysis

**Figure 4:**
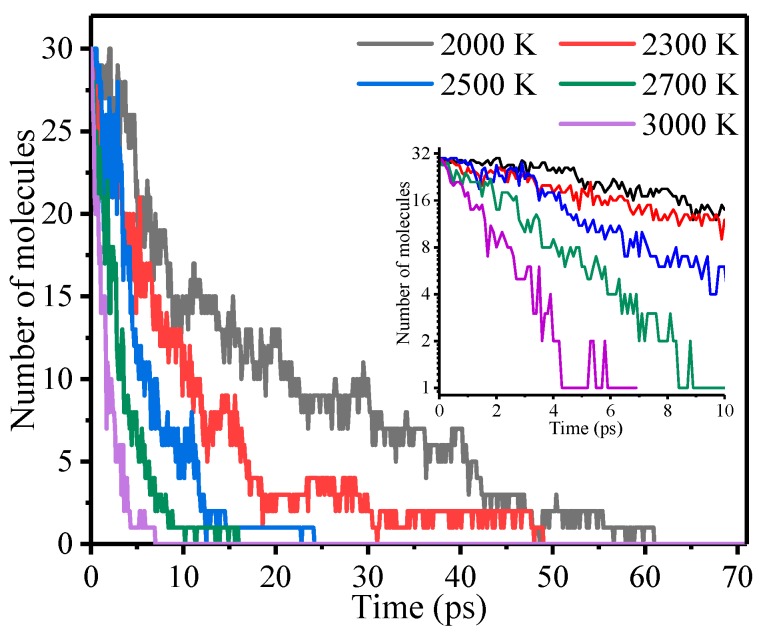
The number of meta-aramid fiber molecules at different temperatures.

The variation in the number of meta-aramid fiber molecules at different temperatures is shown in Figure 4. The number of meta-aramid fibers fluctuated with the progress of thermal decomposition owing to the recombination of some of the broken bonds, which is connected to diffusion [44,45]. At 2000 K, 2300 K, 2500 K, 2700 K, and 3000 K, the total decomposition time of meta-aramid fiber molecules was approximately 60.9, 48.9, 24.1, 15.9, and 6.9 ps, respectively. At higher temperature, the number of meta-aramid fibers decreased more rapidly. With increasing temperature, the decomposition rate increased and the decomposition time decreased because increasing temperature increases the molecular kinetic energy and potential energy.

The thermal cracking kinetics of meta-aramid fibers was evaluated. In the initial decomposition reaction stage, the initial reaction rate (*k*) of the meta-aramid fibers at different temperatures can be obtained using Formula (1) to fit the change in the number of meta-aramid fibers with time [24]:

ln*N*_0_ − ln*N*_t_ = *kt*, (1)

where *t* is the initial decomposition time of the meta-aramid fibers, *N*_0_ is the initial number of meta-aramid fibers, and *N*_t_ is the number of meta-aramid fibers at time *t*.

**Figure 5:**
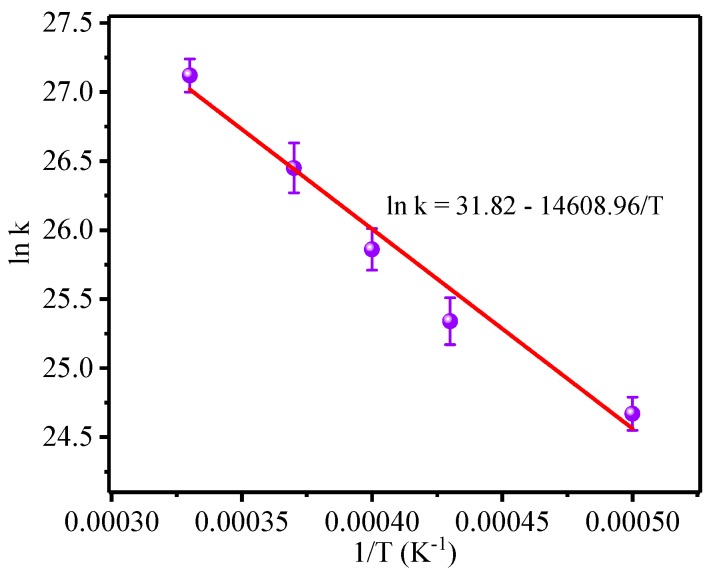
Arrhenius model for meta-aramid fibers.

The relationship between the reaction rate k and temperature was linearly fitted, and the activation energy and pre-exponential factor of the reaction were calculated using an Arrhenius Equation (2) [39]. Table 2 lists the fitting parameters of the Arrhenius equation, and the results of the model fitting are presented in Figure 5.

*k* = *A · *exp(−*E*_a_/*RT*) (2)

Here, *A* is the pre-exponential factor and *E*_a_ is the activation energy.

From the linear fitting results, the slope and intercept were −14,608.96 and 31.82, respectively. Using Formula (2), the activation energy of the meta-aramid fiber was calculated to be 121.45 kJ/mol, and the pre-exponential factor was calculated to be 6.59 × 10^13^.

### 3.3. Statistical Analysis of Major Thermal Decomposition Products

**Figure 6:**
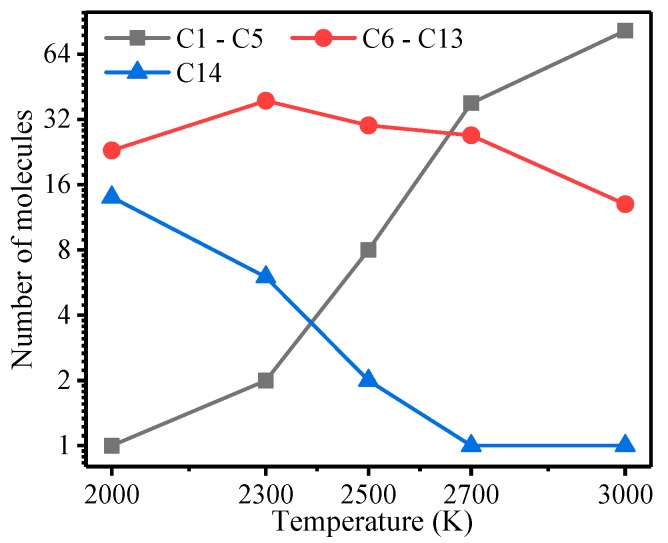
Product of meta-aramid fiber pyrolysis after 100 ps at different temperatures.

The final products of meta-aramid fiber decomposition after 100 ps at different temperatures are shown in Figure 6. Among them, C14 is the unbroken meta-aramid fiber chain with 14 carbon atoms and C6–C13 is the intermediate product of the broken amide bond with 6–13 carbon atoms in the main chain. C1–C5 is the final product of the meta-aramid fiber including the thermal decomposition product of the benzene ring, the product, and the secondary reaction products of the broken amide group. The composition was complex, consisting of many different products with 1–5 carbon atoms in the main chain.

As shown in Figure 6, the number of C14 decreased with increasing temperature. Above 2500 K, the meta-aramid fiber was almost completely decomposed after 100 ps in the dynamics simulation. The number of C6–C13 products showed an upward trend before declining, reaching a maximum at 2300 K. This trend indicates that high temperatures accelerate the thermal decomposition of C6–C13 products. As the temperature increases, the number of C1–C5 products increases, and the increase was almost linear above 2500 K. The correlation between the C6–C13 and C1–C5 products was analyzed. The Poisson correlation between the C6–C13 and C1–C5 products was −0.992, with a significance of 0.008 (far less than 0.05), which indicates a significant correlation between the two variables. This finding suggests that the C1–C5 products are mainly derived from the decomposition of C6–C13 products.

**Figure 7:**
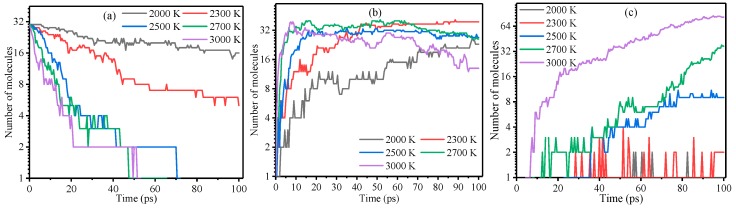
Pyrolysis composition of meta-aramid fiber at different temperatures: (**a**) C14, (**b**) C6–C13, and (**c**) C1–C5 products.

Figure 7 shows the composition of the pyrolysis products of the meta-aramid fiber at different temperatures. The number of C6–C13 rapidly increased as the thermal decomposition proceeded and reached a peak before declining, and the peak time decreased with increasing temperature. With increasing temperature, the times to reach this peak were shortened. From 2000 K to 3000 K, these times for C6–C13 decreased as 97.1, 48.6, 36.5, 16.7, and 9.4 ps. The Poisson correlation between temperature and the C6–C13 peak times was −0.954, which indicates that the relationship between temperature and C6–C13 peak times is significant. As the decomposition proceeded, the number of C1–C5 products continuously increased. A higher temperature resulted in a faster rate of increase. The number of C1–C5 products increased with increasing temperature. The number of C1–C5 products was only 1 after 100 ps of thermal decomposition at a low temperature (less than 2300 K). The main pyrolysis steps for the meta-aramid fiber may be as follows: the amide bond breaks into C6–C13 products, the benzene ring opens, and then C6–C13 products further decompose into C1–C5 products. Segments with long carbon chains further decomposed into products with short carbon chains. The main products of this process were gases (such as methane and acetylene) and free radicals.

**Figure 8:**
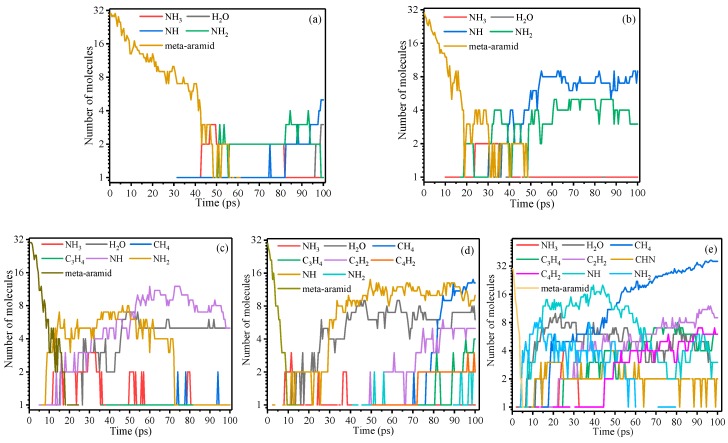
Micromolecular products of meta-aramid fiber at different temperatures: (**a**) 2000 K, (**b**) 2300 K, (**c**) 2500 K, (**d**) 2700 K, and (**e**) 3000 K.

The statistical results of the main pyrolysis products of meta-aramid fiber at different temperatures are presented in Figure 8. At low temperature, the meta-aramid fiber decomposed slowly, and the number of small molecules and free radicals (mainly NH_2_ and NH) produced by thermal cracking was smaller. The initial stage of thermal decomposition was mainly dehydrogenation and C–N bond breaking. These phenomena indicate that the C–N bonds are weak links in the meta-aramid fiber compared with chemical bonds at other positions and are more vulnerable to fracture. When the temperature rises, the reaction rate of the thermal decomposition of the aramid fiber is accelerated, and the types and amounts of the generated products (radicals and small molecules) are also increasing. The free radicals in the model increase with increasing temperature because of the presence of free volume in the system, and the free radicals produced by thermal decomposition flood the space.

The micromolecular products in the thermal decomposition process at the initial time were NH_3_ and H_2_O followed by these products and other carbon-containing micromolecules as the temperature was further increased. With increasing temperature, the initial time at which various micromolecules appeared became shorter and shorter, and almost no change in the quantity of NH_3_ was observed. The quantity of H_2_O molecules increased and showed a relatively stable trend as the temperature increased. In addition, the number of CH_4_, C_2_H_2_, C_3_H_4_, and other hydrocarbon micromolecules increased with increasing temperature. In total, the quantity of CH_4_ products was much higher than that of C2 and C3 hydrocarbons because the generation energy of CH_4_ is lower than those of C2 and C3 [46]. The number of CH_4_ molecules increased dramatically with increasing temperature. After 100 ps, the amount of methane at 3000 K was approximately three times that at 2700 K and approximately 12 times that at 2500 K. Because the number of carbon atoms in the system is set, the amount of methane will eventually stabilize. Figure 8 shows that increasing the temperature increases the number of different products generated in the system, including some complex species; for example, HCN appears at 3000 K and does not appear at other temperatures. Previous studies have shown that stable products such as HCN are only produced at high temperatures [3]. Moreover, there was a small amount of CO_2_, H_2_, and other gases produced at high temperature. Therefore, HCN is a representative gas of meta-aramid pyrolysis and can be used to detect thermal faults.

### 3.4. Generation Mechanism of NH_3_ and H_2_O

A labeling method that marked different elements with different colors was adopted to monitor the reaction process, and the generation path of the products was traced. As the thermal decomposition process proceeded, large quantities of NH_2_, NH, C_2_H, and C_3_H_3_ free radicals were generated. These free radicals accelerated the dehydrogenation of the meta-aramid fiber, producing micromolecules such as NH_3_, C_2_H_2_, C_3_H_4_, and H_2_O. NH_3_ and H_2_O were the first major products in the thermal decomposition process. In this paper, the temperature of 2000 K was used as an example to illustrate the thermal decomposition reaction of the meta-aramid fiber and the generation paths of the NH_3_ and H_2_O micromolecules. For convenience, pink signifies an O atom, yellow signifies an H atom, and light blue signifies an N atom.

**Figure 9:**
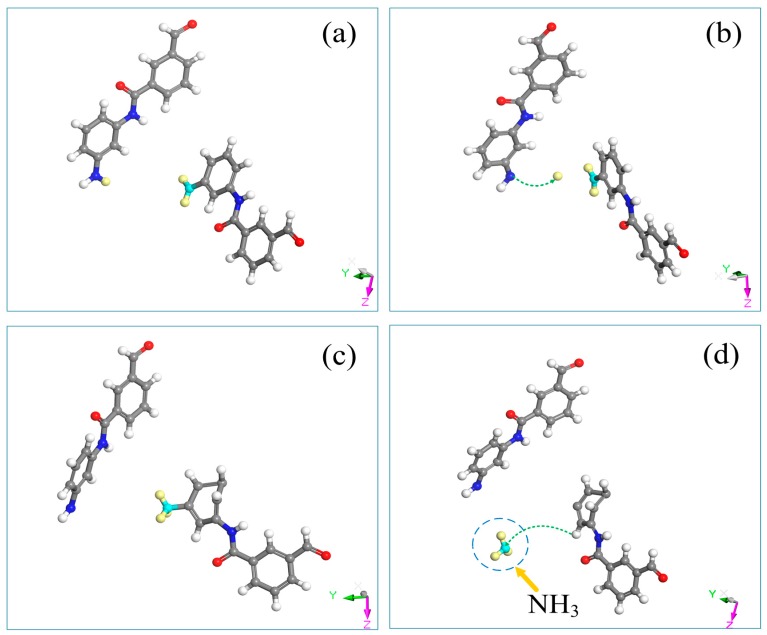
Generation path snapshots of the NH_3_ micromolecule for decomposition at 2000 K: (**a**) 1 ps, (**b**) 21.8 ps, (**c**) 40.9 ps, (**d**) 41.2 ps.

**Figure 10:**
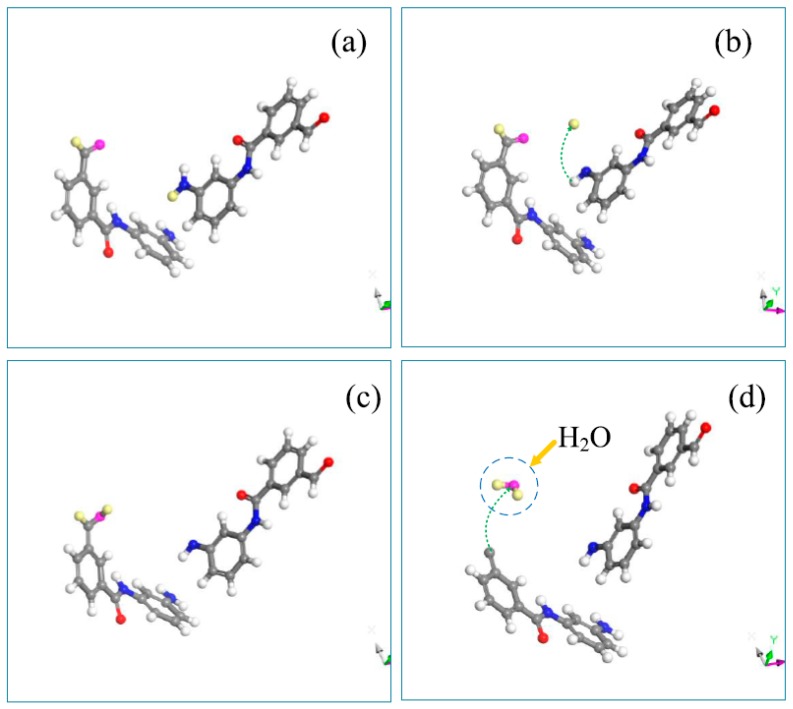
Generation path snapshots of H_2_O micromolecule under decomposition at 2000 K: (**a**) 30.1 ps, (**b**) 48.1 ps, (**c**) 48.4 ps, and (**d**) 52.8 ps.

By tracing the thermal decomposition reaction, ammonia molecules were mainly formed as an NH_2_ molecule captured the adjacent H atom and broke away from the main chain to form NH_3_. Another reaction could be an NH molecule combining with the systematic H atom to form NH_2_, which then captured the adjacent H atom to form NH_3_. Figure 9 shows the generation path of NH_3_ gas. At 21.8 ps, the H atom dissociated from the main chain of the meta-aramid fiber to form a free H atom. At 48.1 ps, NH_2_ at the end of the molecular chain combined with the free H atom and dissociated from the main chain to form NH_3_. The formation of water molecules occurred later in the process. Water molecules were mainly generated from the formation of a hydroxyl group through oxidation and hydrogenation of a carbonyl C=O and then dehydroxylation or intramolecular dehydration. The alternative step could be a carbonyl hydrogenated to form a hydroxyl group, which was then separated from the main chain as OH. This OH attacked an adjacent H atom to generate H_2_O; this mechanism has been previously reported [2]. Figure 10 shows the generation path diagram of water during the pyrolysis of the meta-aramid fiber. At 48.4 ps, a carbonyl group was formed through hydrogen abstraction. At 52.8 ps, an intramolecular elimination reaction occurred during the formation of water.

In addition to NH_3_ and H_2_O, CH_4_ was a major product observed in large quantities, and the generation mechanism of CH_4_ was relatively simple. The main process was the demethylation reaction. In a different route, a CH_2_ group in the system produced a CH_3_ group through hydrogen abstraction and then separated as a CH_4_ group.

## 4. Conclusions

The pyrolysis process of meta-aramid fiber at high temperatures was studied using ReaxFF. The thermal decomposition mechanism of the meta-aramid fiber and potential generation paths of NH_3_, H_2_O, and other products were described on the atomic scale.

The initially broken bond position of the meta-aramid fiber indicates that the C_ar_–N bond is the most frequently broken bond, followed by the C=O double bond. This finding suggests that C_ar_–N bonds are the weak link in meta-aramid fibers and is more vulnerable to fracture. The strengthening of the C_ar_–N bond should thus be given priority for the prevention of meta-aramid fiber aging.

Investigation of the thermal cracking kinetics of the meta-aramid fiber revealed that the activation energy and pre-exponential factor of the meta-aramid fiber were 121.45 kJ/mol and 6.59 × 10^13^, respectively, in the temperature range of 2000–300 K.

The main pyrolysis process of meta-aramid fiber forms C6–C13 products by C_ar_–N bond fracture, and C6–C13 products decompose into fragments of micromolecules and gases. Temperature is the key factor affecting the thermal decomposition of the meta-aramid fiber, and increasing the temperature will accelerate the thermal decomposition rate. Gas species in the model become more complex with increasing temperature. CH_4_ increases as the systematic pyrolysis energy increases, and high temperature is conducive to producing more stable gases. HCN is a representative gas of high-temperature pyrolysis. For application, these micromolecular gases can be tested using spectroscopic analysis and used for diagnosis and detection of overheating faults.

At different temperatures, NH_3_ and H_2_O are the first products. The corresponding generation paths indicate that the main origin of micromolecular NH_3_ is when the NH_2_ at the meta-aramid fiber terminal captures an H atom adjacent to the system. H_2_O is formed as the carbonyl group becomes a hydroxyl group through oxidation and hydrogenation, and then dehydroxylation or intramolecular dehydration occurs.

## Figures and Tables

**Table 1:** Statistical results of the initially broken bond fractures for the 3-PMIA model.

Chemical bond	C_ar_–N	C_aromatic ring_-C_carbonyl_	C=O	Other
**Broken times**	15	1	10	4
**Proportion (%)**	50	3	33	14

**Table 2:** Fitting parameters of Arrhenius model.

***T*** **(K)**	2000	2300	2500	2700	3000
**1/*T*(K^−1^)**	0.00050	0.00043	0.00040	0.00037	0.00033
**ln*k***	24.67	25.34	25.86	26.45	27.12

## References

[1] Wen M.M., Tian M.Q., Song Y., Liu Y., Lei Z.P., Song J.C. (2014). Aging Law and Reliability Analysis of Nomex Paper Used for Dry-type Transformer Insulation. High Volt. Eng..

[2] Schulten H.R., Plage B., Ohtani H., Tsuge S. (1987). Studies on the thermal degradation of aromatic polyamides by pyrolysis-field ionization mass spectrometry and pyrolysis-gas chromatography. Angew. Makromol. Chem..

[3] Wang X.W., Hu Z.M., Liu Z.F. (2008). Study on the Pyrolytic Decomposition Process of Meta- and Para-Aramid Fibers by Py/GC-MS & TGA-DTA/MS. J. Instrum. Anal..

[4] Mettler M.S., Mushrif S.H., Paulsen A.D., Javadekar A.D., Vlachos D.G., Dauenhauer P.J. (2012). Revealing pyrolysis chemistry for biofuels production: Conversion of cellulose to furans and small oxygenates. Energy Environ. Sci..

[5] Agarwal V., Dauenhauer P.J., Huber G.W., Auerbach S.M. (2012). Ab initio dynamics of cellulose pyrolysis: Nascent decomposition pathways at 327 and 600 °C. J. Am. Chem. Soc..

[6] Warshel A., Weiss R.M. (2010). Empirical valence bond calculations of enzyme catalysis. Ann. N. Y. Acad. Sci..

[7] Nagy T., Yosa R.J., Meuwly M. (2014). Multisurface Adiabatic Reactive Molecular Dynamics. J. Chem. Theory Comput..

[8] Brickel S., Meuwly M. (2017). OH-Stretching Overtone Induced Dynamics in HSO3F from Reactive Molecular Dynamics Simulations. J. Phys. Chem. A.

[9] Shi X., Xiao H., Lackner K.S., Chen X. (2016). Capture CO_2_ from Ambient Air Using Nanoconfined Ion Hydration. Angew. Chem. Int. Ed. Engl..

[10] Eslami H., Bahri K., MüllerPlathe F. (2018). Solid−Liquid and Solid−Solid Phase Diagrams of Self-Assembled Triblock Janus Nanoparticles from Solution. J. Phys. Chem. C.

[11] Hu J.Y., Liu C., Li Q.B., Shi X.Y. (2018). Molecular simulation of thermal energy storage of mixed CO_2_/IRMOF-1 nanoparticle nanofluid. Int. J. Heat Mass Transf..

[12] Van Duin A.C.T., Dasgupta S., Lorant F., Goddard W.A. (2001). ReaxFF: A reactive Force Field for Hydrocarbons. J. Chem. Phys..

[13] Zhang X.X., Wu Y.J., Chen X.Y., Wen H., Xiao S. (2017). Theoretical Study on Decomposition Mechanism of Insulating Epoxy Resin Cured by Anhydride. Polymers.

[14] Strachan A., van Duin A.C.T., Chakraborty D., Dasgupta S., Goddard W.A. (2003). Shock waves in high-energy materials: The initial chemical events in nitramine RDX. Phys. Rev. Lett..

[15] Nielson K.D., van Duin A.C.T., Oxgaard J., Deng W.Q. (2005). Development of the ReaxFF reactive force field for describing transition metal catalyzed reactions, with application to the initial stages of the catalytic formation of carbon nanotubes. J. Phys. Chem. A.

[16] Kulkarni A.D., Truhlar D.G., Srinivasan S.G., van Duin A.C.T., Norman P., Schwartzentruber T.E. (2015). Oxygen Interactions with Silica Surfaces: Coupled Cluster and Density Functional Investigation and the Development of a New ReaxFF Potential. J. Phys. Chem. C.

[17] Chenoweth K., van Duin A.C.T., Goddard W. (2009). The ReaxFF Monte Carlo reactive dynamics method for predicting atomistic structures of disordered ceramics: Application to the Mo_3_VO_x_ catalyst. Angew. Chem. Int. Ed. Engl..

[18] Zandiatashbar A., Lee G.H., An S.J., Lee S., Mathew N., Terrones M., Hayashi T., Picu C.R., Hone J., Koratkar N. (2014). Effect of defects on the intrinsic strength and stiffness of graphene. Nat. Commun..

[19] Lin L.C., Grossman J.C. (2015). Atomistic understandings of reduced graphene oxide as an ultrathin-film nanoporous membrane for separations. Nat. Commun..

[20] Zhang C., Wen Y., Xue X. (2014). Self-enhanced catalytic activities of functionalized graphene sheets in the combustion of nitromethane: Molecular dynamic simulations by molecular reactive force field. ACS Appl. Mater. Interfaces.

[21] Beste A. (2014). ReaxFF Study of the Oxidation of Lignin Model Compounds for the Most Common Linkages in Softwood in View of Carbon Fiber Production. J. Phys. Chem. A.

[22] Liu X.L., Li X.X., Han S., Qiao X.J., Zhong B.J., Guo L. (2016). Initial Reaction Mechanism of RP-3 High Temperature Oxidation Simulation With ReaxFF MD. Acta Phys. Chim. Sin..

[23] Cheng T., Jaramillobotero A., Sun H. (2014). Adaptive Accelerated ReaxFF Reactive Dynamics with Validation from Simulating Hydrogen Combustion. J. Am. Chem. Soc..

[24] Rom N., Zybin S.V., van Duin A.C.T., Goddard W.A., Zeiri Y., Katz G., Kosloff R. (2011). Density-dependent liquid nitromethane decomposition: Molecular dynamics simulations based on ReaxFF. J. Phys. Chem. A.

[25] Page A.J., Moghtaderi B. (2009). Molecular Dynamics Simulation of the Low-Temperature Partial Oxidation of CH_4_. J. Phys. Chem. A.

[26] Wang X.L., Li Q.M., Zhang Y., Yang R., Gao S.G. (2017). Simulation of Reactive Molecular Dynamics of Transformer Oil Pyrolysis at High Temperature and the Influence Mechanism of Acid in Oil. High Volt. Eng..

[27] Yan J.Y., Wang X.L., Li Q.M., Zhou Y., Wang Z.D., Li C.G. (2015). Molecular Dynamics Simulation on the Pyrolysis of Insulating Paper. Proc. CSEE.

[28] Shi L., Zhao T., Shen G., Hou Y., Zou L., Zhang L. Molecular dynamics simulation on generation mechanism of water molecules during pyrolysis of insulating paper. Proceedings of the IEEE International Conference on High Voltage Engineering and Application.

[29] Castro-Marcano F., Russo M.F., van Duin A.C.T., Mathews J.P. (2014). Pyrolysis of a large-scale molecular model for Illinois no. 6 coal using the ReaxFF reactive force field. J. Anal. Appl. Pyrol..

[30] Jain A., Vijayan K. (2002). Thermally induced structural changes in Nomex fibres. Bull. Mater. Sci..

[31] Varini N., English N.J., Trott C.R. (2012). Molecular Dynamics Simulations of Clathrate Hydrates on Specialised Hardware Platforms. Energies.

[32] Tang C., Li X., Li Z.W., Hao J. (2017). Interfacial Hydrogen Bonds and Their Influence Mechanism on Increasing the Thermal Stability of Nano-SiO_2_-Modified Meta-Aramid Fibres. Polymers.

[33] Zha W., Song H.T., Dang Z.M., Shi C.Y., Bai J.B. (2008). Mechanism analysis of improved corona-resistant characteristic in polyimide/TiO_2_ nanohybrid films. Appl. Phys. Lett..

[34] Dong M., Wang H., Shen L., Ye Y., Ye C., Wang Y., Zhang J., Jiang Y. (2012). Dielectric property and electrical conduction mechanism of ZrO_2_–TiO_2_ composite thin films. J. Mater. Sci. Mater. Electron..

[35] Yin F., Tang C., Li X., Wang X.B. (2017). Effect of Moisture on Mechanical Properties and Thermal Stability of Meta-Aramid Fiber used in Insulating Paper. Polymers.

[36] Zha J.W., Meng X., Wang D.R., Dang Z.M., Li R.K.Y. (2014). Dielectric properties of poly(vinylidene fluoride) nanocomposites filled with surface coated BaTiO_3_ by SnO_2_ nanodots. Appl. Phys. Lett..

[37] Bourbigot S., Flambard X. (2010). Heat resistance and flammability of high performance fibres: A review. Fire Mater..

[38] Zhang S.F. (2009). Correlation between the Interface and Structure Characteristics of Meta-Aramid Fiber and the Properties for Sheetmaking. Ph.D. Thesis.

[39] Ding J., Zhang L., Zhang Y., Han K.L. (2013). A reactive molecular dynamics study of n-heptane pyrolysis at high temperature. J. Phys. Chem. A.

[40] Yin K.L., Wu G.Y., Chen C.L. (2001). Investigation of Thermal Cracking Process of *n*-Decane by Molecular Dynamics Simulation. Acta Pet. Sin..

[41] Mayo S.L., Olafson B.D., Goddard W.A. (1990). DREIDING: A generic force field for molecular simulations. J. Phys. Chem..

[42] Zhang L., Chen L., Wang C., Wu J.Y. (2013). Molecular Dynamics Study of the Effect of H_2_O on the Thermal Decomposition of α Phase CL-20. Proc. CSEE.

[43] Chen B., Diao Z.J., Lu H.Y. (2014). Using the ReaxFF reactive force field for molecular dynamics simulations of the spontaneous combustion of lignite with the Hatcher lignite model. Fuel.

[44] Eslami H., Heydari N. (2013). Hydrogen bonding in water nanoconfined between graphene surfaces: A molecular dynamics simulation study. J. Nanopart. Res..

[45] Afandak A., Eslami H. (2017). Ion-Pairing and Electrical Conductivity in the Ionic Liquid 1-*n*-Butyl-3-methylimidazolium Methylsulfate [Bmim][MeSO_4_]: Molecular Dynamics Simulation Study. J. Phys. Chem. B.

[46] Zhang Y. (2016). Research on Micro-Mechanism of Pyrolysis Process of Oil-Paper Insulation in Power Transformer. Master’s Thesis.

